# Synergistic oxidation of toluene through bimetal/cordierite monolithic catalysts with ozone

**DOI:** 10.1038/s41598-024-58026-6

**Published:** 2024-03-26

**Authors:** Xiaojian Wang, Xiaomin Peng, Quanzhong Zhao, Jinxing Mi, Huating Jiang, Shengli Li, Hui Hu, Hao Huang

**Affiliations:** 1 Shanghai Tobacco Group Co. LTD, Shanghai, 200082 People’s Republic of China; 2 School of Environmental Science and Engineering, Huazhong University of Science and Technology, Wuhan, 430074 People’s Republic of China; 3 Hubei Key Laboratory of Multi-Media Pollution Cooperative Control in Yangtze Basin, School of Environmental Science and Engineering, Huazhong University of Science and Technology, Wuhan, 430074 People’s Republic of China; 4 Inner Mongolia Power Research Institute Branch, Inner Mongolia Power (Group) Co., Ltd., Hohhot, 010020 People’s Republic of China; 5 State Key Joint Laboratory of Environment Simulation and Pollution Control, School of Environment, Tsinghua University, Beijing, 100084 People’s Republic of China

**Keywords:** Pollution remediation, Chemical engineering

## Abstract

Toluene treatment has received extensive attention, and ozone synergistic catalytic oxidation was thought to be a potential method to degrade VOCs (violate organic compounds) due to its low reaction temperature and high catalytic efficiency. A series of bimetal/Cord monolithic catalysts were prepared by impregnation with cordierite, including Mn_x_Cu_5−x_/Cord, Mn_x_Co_5−x_/Cord and Cu_x_Co_5−x_/Cord (x = 1, 2, 3, 4). Analysis of textural properties, structures and morphology characteristics on the prepared catalysts were conducted to evaluate their performance on toluene conversion. Effects of active component ratio, ozone addition and space velocity on the catalytic oxidation of toluene were investigated. Results showed that Mn_x_Co_5−x_/Cord was the best among the three bimetal catalysts, and toluene conversion and mineralization rates reached 100 and 96% under the condition of Mn_2_Co_3_/Cord with 3.0 g/m^3^ O_3_ at the space velocity of 12,000 h^−1^. Ozone addition in the catalytic oxidation of toluene by Mn_x_Co_5−x_/Cord could efficiently avoid the 40% reduction of the specific surface area of catalysts, because it could lower the optimal temperature from 300 to 100 °C. (Co/Mn)(Co/Mn)_2_O_4_ diffraction peaks in XRD spectra indicated all the four Mn_x_Co_1−x_/Cord catalysts had a spinel structure, and diffraction peak intensity of spinel reached the largest at the ratio of Mn:Co = 2:3. Toluene conversion rate increased with rising ozone concentration because intermediate products generated by toluene degradation might react with excess ozone to generate free radicals like ·OH, which would improve the toluene mineralization rate of Mn_2_Co_3_/Cord catalyst. This study would provide a theoretical support for its industrial application.

## Introduction

Volatile organic compounds (VOCs) not only affect the ambient air quality, but also increase health risks to humans, and some polycyclic aromatic hydrocarbons (PAH) even cause cancer^1–3^. The compositions of VOCs emitted from various industries are totally different, and a single treatment process can hardly meet requirements of all the VOCs control^4–6^. Research on finding out a high efficiency, environmentally friendly and economical method with extensive application to control VOCs has always been a hotspot in the field of air pollution control^7–9^. As a typical VOC from both industrial and traffic emissions, toluene has a high ozone formation potential and its treatment has received wide attention^10,11^. Regenerative thermal oxidizer (RTO) and thermal oxidizer (TO) are often used in VOCs treatment including toluene, however adding large amounts of natural gas leads to a poor economy^12–14^. Catalytic combustion is also used to remove toluene with Ce and Mn containing catalysts^15,16^, while restricting the formation of dioxins under low temperature is a problem^17–19^. Discharge plasma is another method to degrade toluene, and various catalysts are used in the synergistic removal with plasma^20–23^, however complex equipment and inconsistent removal efficiency limit its industrial application. Photocatalysis is also thought to be potential method to removal toluene, and it can not only provide hydrogen energy^24–27^, but also mineralize VOCs into CO_2_ and H_2_O with catalysis^28–30^. Electrocatalytic oxidation is an environmentally friendly method in toluene treatment^31,32^, and toluene removal performance with different kinds of catalysts like CeO_2_ has been investigated^33–35^, while the removal efficiency is not high enough under the complex flue gas with large flue rate. Biological methods for the removal of gaseous toluene face with the similar problems^36–38^.

As a typical strong oxidant, ozone is able to oxidize VOCs into the highest valence state. However, reactions between ozone and organic matters often occurs with high ozone concentration when catalysts are absent, and their chemical reaction rates are relatively low. In the ozone catalytic oxidation method, catalysts are utilized to catalyze ozone to generate O^·^ and ·OH radicals, which can efficiently convert VOCs into CO_2_ and H_2_O^39,40^. Screening and preparation of catalysts are the key parts in this method. Cordierite (Cord) is widely used as a catalyst carrier for removing industrial VOCs, due to thermal stability, high specific surface utilization, and stable chemical properties^41^. Some metals and their oxides have attracted attentions due to their good catalytic performance in the toluene degradation^42–45^, and mixed oxides often show a higher degradation rate than a single metal oxide because of the synergistic effect between them^46–48^. Ozone catalytic oxidation technology was used to degrade toluene in this study, and a series of bimetal/Cord monolithic catalysts were prepared, including Mn_x_Cu_5−x_/Cord, Mn_x_Co_5−x_/Cord and Cu_x_Co_5−x_/Cord (x = 1, 2, 3, 4). The effects of active components ratio, ozone concentration and space velocity on toluene degradation rates were systematically investigated. Results of this research would provide an efficient and simple method for toluene degradation by ozone catalytic oxidation, and build a theoretical basis for its future industrial application in VOCs emission control.

## Experimental setup and materials

### Experimental materials

#### Catalyst preparation

Mn(NO_3_)_2_, Co(NO_3_)_2_ and Cu(NO_3_)_2_ (AR, Aladdin, China) were prepared into a mixed solution with a total ion concentration of 1.0 mol/L according to the molar ratio, then the same amount of citric acid (AR, Aladdin, China) was added, and then the cordierite carrier (Haichuan Chemical, China) was immersed in the solution for 30 min. After blowing off the residual solution, it was placed in an oven at 110 °C for 10 h, and then calcined in a muffle furnace at 500 °C for 5 h. Finally, the above steps were repeated once to obtain the catalysts required for the experiment, which were marked as Mn_x_Cu_5−x_/Cord (Mn_1_Cu_4_/Cord, Mn_2_Cu_3_/Cord, Mn_3_Cu_2_/Cord, and Mn_4_Cu_1_/Cord), Mn_x_Co_5−x_/Cord (Mn_1_Co_4_/Cord, Mn_2_Co_3_/Cord, Mn_3_Co_2_/Cord, and Mn_4_Co_1_/Cord) and Cu_x_Co_5−x_/Cord (Cu_1_Co_4_/Cord, Cu_2_Co_3_/Cord, Cu_3_Co_2_/Cord, and Cu_4_Co_1_/Cord), respectively.

#### Catalyst characterization

The BET specific surface area was tested, pore volume, and average pore diameter of the catalyst were obtained using an ASAP-2020 apparatus (Micromeritics, USA). The X-ray diffraction (XRD) patterns (D8 advance, Bruker, Germany) were determined in the range of 10°–80° at a rate of 2°/min by using an X-ray diffractometer with Cu-Kα radiation. The morphology of the catalyst was measured by scanning electron microscope (SEM) (MIRA4, Tescan, Czech), the field emission voltage was 15 kV, and the samples were treated with gold spray before testing. Fourier transform infrared reflection (FTIR) (Nicolet iS20, Thermo Scientific, USA) was used to detect the functional groups on the catalyst surface. The swept wavenumber ranged from 4000 to 400 cm^−1^, and the catalyst samples were firstly sieved and pressed into powder and, and then they were tested.

### Experimental setup

**Figure 1:**
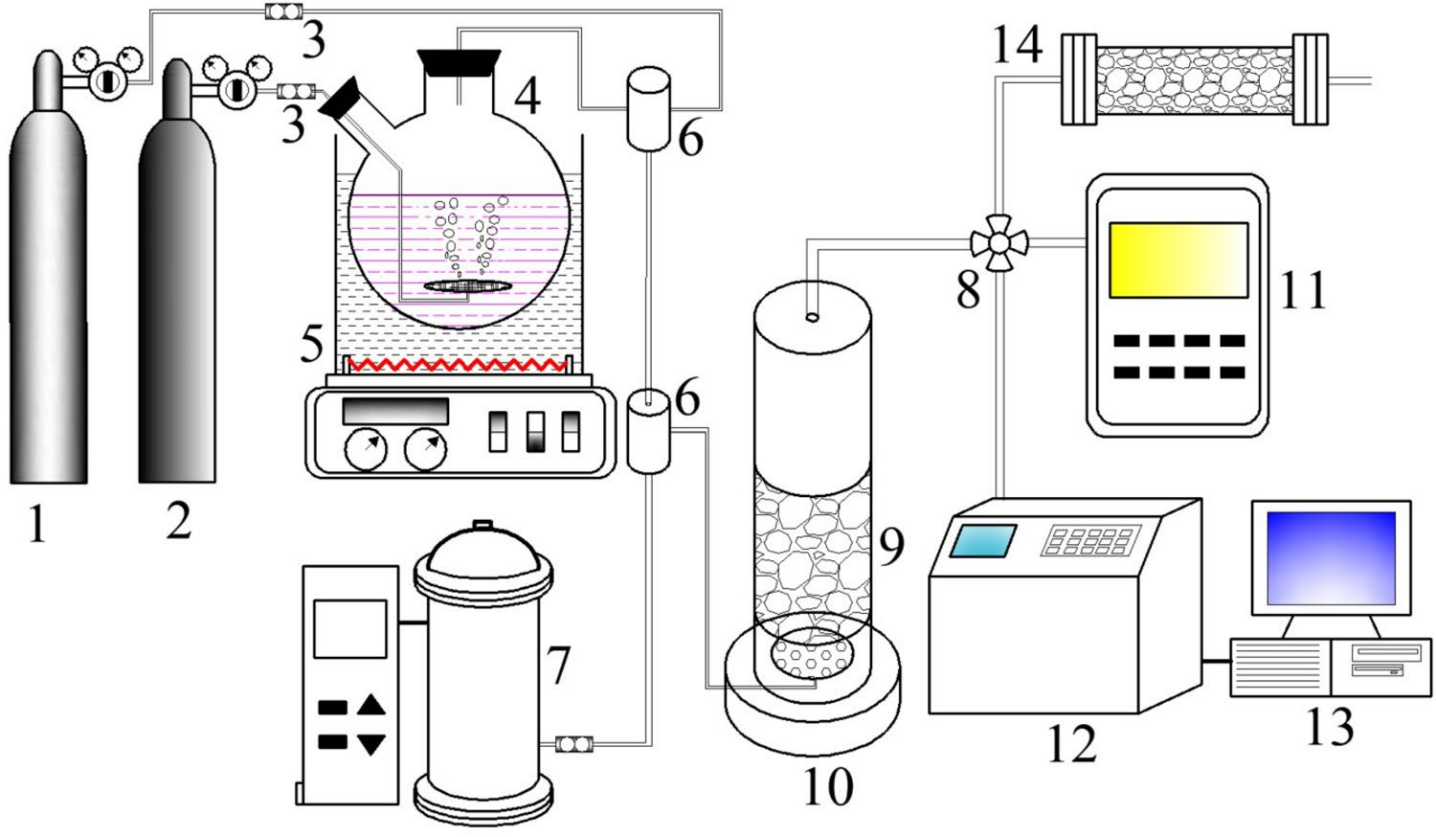
Experimental setup for toluene degradation by bimetal/Cordierite catalyst with ozone (1. Compressed air; 2. N_2_; 3. Gas flow meter; 4. Toluene generator; 5. Thermostat water bath; 6. Mixed gas cylinder; 7. Ozone generator; 8. Three-way valve; 9. Catalysts; 10. Heating chamber; 11. Ozone detector; 12. Gas chromatography; 13. Computer; 14. Activated carbon absorption tower.)

Catalytic reaction experiments were performed in a fixed-bed reactor. The diagram of experimental setup was showed in Fig. 1. N_2_ was used as a carried to blow out the toluene from bubbling bottle in the constant temperature water bath, and the compressed air entered the gas mixing bottle and mixed with the inflowing toluene gas to form different concentrations of toluene gas. The ozone from the ozone generator and the toluene gas in the mixing bottle entered the reactor together with for ozone catalytic oxidation reaction. The concentration of raw materials and tail gas was detected by ozone detector and gas chromatography (HP 6890, Agilent, USA), and the remaining tail gas was discharged after passing through the activated carbon absorption tower. Carbon monoxide was not detected in the experiment, and the carbon dioxide in the tail gas was determined by solution absorption method.

### Evaluation of toluene degradation performance

Toluene conversion and mineralization rates were both used to evaluate the performance of toluene degradation by bimetal/Cord monolithic catalysts with ozone in this study. The toluene conversion rate *η* was defined in Eq. (1).

1 $$\eta = \frac{{c\left( {{\text{C}}_{7} {\text{H}}_{8,in} } \right) - c\left( {{\text{C}}_{7} {\text{H}}_{8,out} } \right)}}{{c\left( {{\text{C}}_{7} {\text{H}}_{8,in} } \right)}} \times 100\%$$

where *C*(C_7_H_8,in_) was the initial concentration of toluene at the inlet, mg/m^3^; *C*(C_7_H_8,in_) was the concentration of toluene at the outlet, mg/m^3^.

Since the simulated air was used as the dilution gas in this experiment, it almost did not contain CO_2_. Meanwhile the whole experiment setups was well sealed. The CO_2_ in the tail gas from the outlet was mainly from toluene degradation. In addition, the results of the gas chromatography on the tail gas showed that it did not contain CO, so the mineralization rate of toluene in this experiment was only for CO_2_. Concentration of CO_2_ in the tail gas was measured by solution titration. The tail gas was passed into an excess of Ba(OH)_2_ solution, and then the remaining Ba(OH)_2_ was titrated with standard ethanedioic acid solution. Finally the CO_2_ concentration was measured by dividing the titration result by the volume of the gas sample. The mineralization rate *θ* could be calculated by Eq. (2).

2 $$\theta = \frac{{c\left( {{\text{CO}}_{2,out} ,{\text{CO}}_{2,in} } \right)}}{{7 \times \left[ {c\left( {{\text{C}}_{7} {\text{H}}_{8,in} } \right) - c\left( {{\text{C}}_{7} {\text{H}}_{8,out} } \right)} \right]}} \times 100\%$$

where *C*(CO_2, in_) was the initial concentration of CO_2_ at the inlet, mg/m^3^; *C*(CO_2, out_) was the concentration of CO_2_ at the outlet, mg/m^3^.

## Results and discussion

### Evaluations on characterization of catalysts

#### Textural properties of the bimetal/cord catalysts

To find out the textural properties of the prepared catalysts, BET specific surface area tests were carried out on the Mn_x_Cu_5−x_/Cord, Mn_x_Co_5−x_/Cord and Cu_x_Co_5−x_/Cord. Results indicated that the BET surface areas of Mn_x_Co_5−x_/Cord was generally bigger than those of Mn_x_Cu_5−x_/Cord and Cu_x_Co_5−x_/Cord, which was shown in Table s1 and s2 in the supporting information. Textural properties of Mn_x_Co_5−x_/Cord were listed in Table 1, including before and after catalytic oxidation reactions as well as with and without ozone.

**Table 1:** Textural properties of the catalysts.

Samples	a: Before reaction b: After reaction without ozone c: After reaction with ozone	BET surface area (m^2^/g)	Pore volume (cm^3^/g)	Average pore diameter (nm)
Cord	a	1.0621	0.0030	11.926
Mn_1_Co_4_/Cord	a	6.5071	0.0154	8.6700
	b	3.8129	0.0105	10.2760
	c	6.7032	0.0160	8.8564
Mn_2_Co_3_/Cord	a	10.1179	0.0222	8.5710
	b	5.3717	0.0128	9.5392
	c	10.1063	0.0220	8.5364
Mn_3_Co_2_/Cord	a	7.5485	0.0176	6.5262
	b	4.8054	0.0119	9.7304
	c	7.4387	0.0173	6.4965
Mn_4_Co_1_/Cord	a	9.0851	0.0178	7.6135
	b	5.2623	0.0123	9.2196
	c	9.0749	0.0179	7.6097

The specific surface area of the blank cordierite carrier was small, only 1.0621 m^2^/g. When the active components were loaded, the specific surface area of the catalyst increased significantly, and the top BET surface area of Mn_x_Co_5−x_/Cord reached 10.1179 m^2^/g at the ratio of Mn:Co = 2:3, which was much bigger than 3.2202 m^2^/g at the ratio of Mn:Cu = 2:3 and 3.8408 m^2^/g at the ratio of Co:Cu = 2:3 as show in Table s1 and s2. Due to the loading of different proportions of active components, the specific surface area of the catalyst was different. The overall size order of the BET surface area was Mn_x_Co_5−x_/Cord > Cu_x_Co_5−x_/Cord > Mn_x_Cu_5−x_/Cord. Among the four Mn_x_Co_5−x_/Cord (x = 1, 2, 3, 4), the size order before reactions was Mn_2_Co_3_/Cord > Mn_4_Co_1_/Cord > Mn_3_Co_2_/Cord > Mn_1_Co_4_/Cord. When ozone was not added, the specific surface area of the catalyst after the reaction was smaller than that of the catalyst before the reaction, which was reduced by around 40%. This was mainly because the reaction temperature required for catalytic combustion was high. Catalysts were sintered and agglomerated, forming larger particles and blocking the pores at high temperature, resulting in the reduction of the specific surface area. After adding ozone, the reaction temperature required for catalytic combustion was greatly decreased, and high-temperature sintering of the catalyst could be efficiently avoided, thus the specific surface area after adding ozone reaction did not show an obvious change.

#### Structure characteristics of the bimetal/cord catalysts

**Figure 2:**
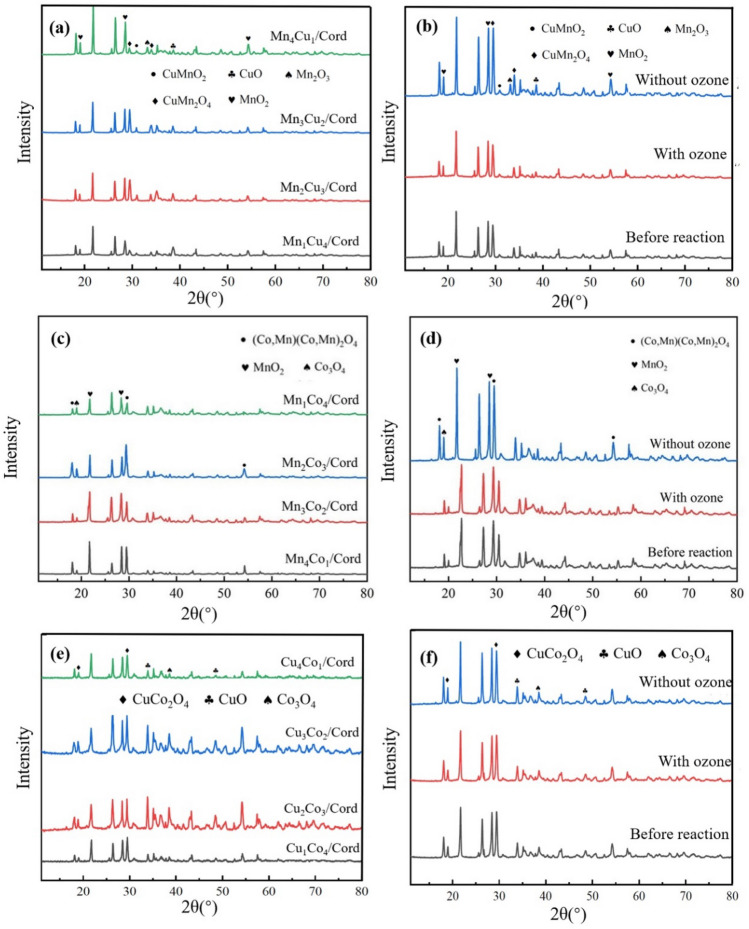
(**a**) XRD of Mn_x_Cu_5−x_/Cord; (**b**) Mn_2_Cu_3_/Cord before and after reactions; (**c**) XRD of Mn_x_Co_5−x_/Cord; (**d**) Mn_2_Co_3_/Cord before and after reactions; (**e**) XRD of Cu_x_Co_5−x_/Cord; (**f**) Cu_3_Co_2_/Cord before and after reactions.

To investigate the structure characteristics of the prepared catalysts, XRD tests on the Mn_x_Cu_5−x_/Cord, Mn_x_Co_5−x_/Cord and Cu_x_Co_5−x_/Cord were conducted both before and after catalytic oxidation reactions as well as with and without ozone. The results were shown in Fig. 2.

XRD of catalysts with different bimetal ratios were shown in Fig. 2a, c and e. Taking Mn_x_Co_5−x_/Cord for instance, all four catalysts had Co_3_O_4_ diffraction peak at 2θ = 19.1°, and its intensity increased with the rising Co content. The MnO_2_ diffraction peaks are at 2θ = 21.8° and 28.5°, and their intensity increased with the rising amount of Mn. At 2θ = 18.1°, 29.5°, and 54.3°, there were (Co/Mn)(Co/Mn)_2_O_4_ diffraction peaks, indicating that all the four catalysts had a spinel structure^49^. In addition, the crystal phase structure of the catalyst did not change with the Mn/Co ratio. Mn and Co showed the same chemical valence state and similar ionic radius, which was conducive to the formation of Mn/Co spinel. When Mn: Co was 2:3, the diffraction peak intensity of spinel reached the largest, thus the highest toluene degradation efficiency was considered to be obtained.

The changing XRD of Mn_2_Cu_3_/Cord, Mn_2_Co_3_/Cord and Cu_3_Co_2_/Cord monolithic catalyst before and after reactions could be seen in Fig. 2b, d and f. Whether ozone was added or not, the overall structure of the catalyst did not change before and after the catalytic oxidation reactions. When ozone was not added, the crystallinity of the catalyst after the reaction increased. However after adding ozone, the XRD of the catalyst before and after the reaction did not change significantly. This was mainly because the number of active sites provided by the catalyst decreased with its reducing specific surface area. This inhibited the increase of catalyst activity and lead to an increase in catalyst crystallinity^50^, which was also consistent with the results of BET tests.

#### Morphology characteristics of the bimetal/cord catalysts

**Figure 3:**
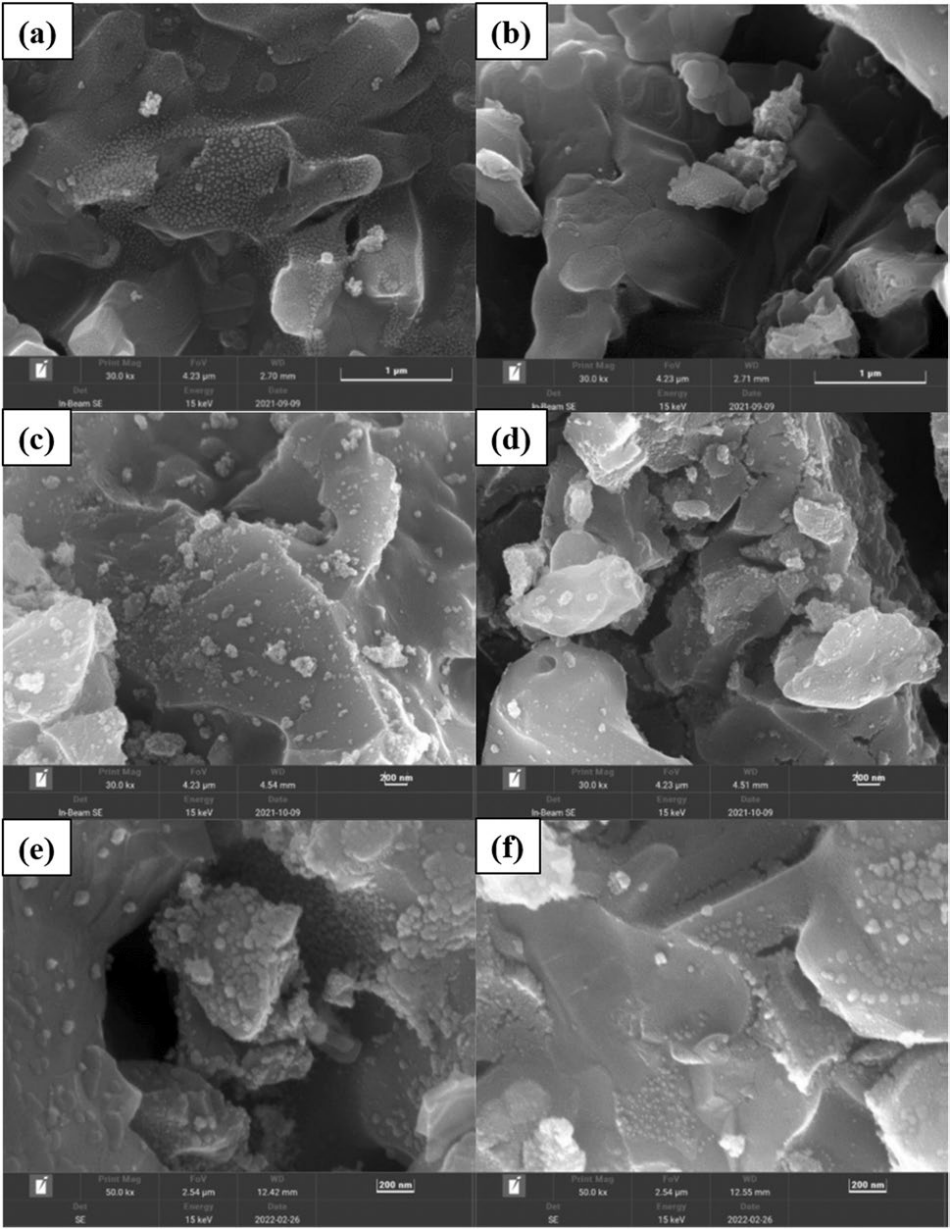
SEM images of bimetal/Cord catalysts after toluene oxidation: (**a**) Mn_2_Cu_3_/Cord without ozone; (**b**) Mn_2_Cu_3_/Cord with ozone; (**c**) Mn_2_Co_3_/Cord without ozone; (**d**) Mn_2_Co_3_/Cord with ozone; (**e**) Cu_3_Co_2_/Cord without ozone; (**f**) Cu_3_Co_2_/Cord with ozone.

Morphology characteristics of Mn_x_Cu_5−x_/Cord, Mn_x_Co_5−x_/Cord and Cu_x_Co_5−x_/Cord monolithic catalysts before catalytic oxidation reactions were investigated by SEM. Mn_2_Cu_3_/Cord, Mn_2_Co_3_/Cord and Cu_3_Co_2_/Cord catalysts after reactions with and without ozone were tested as well. SEM images were shown in Fig. 3.

After loading the active component, the surface of three bimetal/Cord catalysts in Fig. 3 was rough and formed a layer of homogeneous and dense crystal structure when compared with the SEM of blank cordierite carrier in Fig. s1, which indicated that the active component was successfully loaded on the surface of carrier. The SEM of Mn_2_Cu_3_/Cord after catalytic oxidation of toluene with and without ozone were shown in Fig. 3b and a. The size of the particulates without ozone were larger than those after ozone-catalyzed oxidation, which suggested that agglomeration of particulate matter did occur under high temperature. This clogged up the pores and leaded to a decrease in the specific surface area of the catalysts. This result was consistent with BET results in Table s1. SEM image of Mn_2_Co_3_/Cord catalyst after catalytic oxidation reactions with and without ozone was shown in Fig. 3d and c. Both of them were coarser than the blank cordierite shown in Fig. s1. The particulate size on the surface of Mn_2_Co_3_/Cord without ozone was larger than that with ozone, which indicated that the particulates also agglomerated under high temperature, thus blocking the pores, resulting in the reduction of the specific surface area of the catalyst. This result was also consistent with the previous BET results. Figure 3e and f showed the SEM of Cu_3_Co_2_/Cord without and with ozone, respectively. The size of the particulates after the reaction without the addition of ozone were larger than those with ozone, and the number of particulates was also bigger on the high side, which was consistent with the results obtained by XRD, suggesting that the crystallization degree of the catalysts increased after the addition of ozone.

#### FTIR of the bimetal/cord catalysts

**Figure 4:**
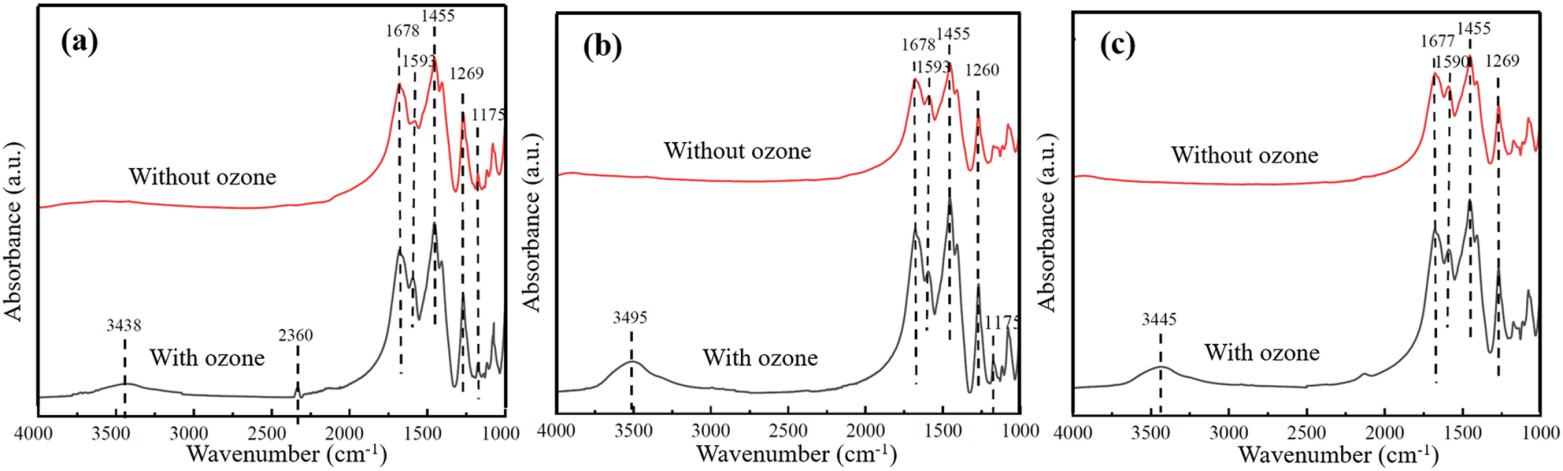
FTIR of bimetal/Cord catalysts (**a**) Mn_2_Cu_3_/Cord; (**b**) Mn_2_Co_3_/Cord; (**c**) Cu_3_Co_2_/Cord.

In order to detect the functional groups on the catalyst surface and find out the pathways of toluene degradation, FTIR of Mn_2_Cu_3_/Cord, Mn_2_Co_3_/Cord and Cu_3_Co_2_/Cord monolithic catalysts were tested. Results were shown in Fig. 4.

The FTIR spectra of Mn_2_Cu_3_/Cord catalyst was shown in Fig. 4a including the catalytic oxidation of toluene with and without ozone. Results showed a bending vibration of the C–H bond on the benzene ring was observed at an absorption wavelength of 1175 cm^−1^, and the vibration peak of benzene ring skeleton from toluene was observed at 1455 cm^−1^^51^. This suggested that toluene was indeed adsorbed on the surface of the Mn_2_Cu_3_/Cord catalysts. The benzene ring vibration peaks of the catalysts turned to be stronger with the addition of ozone, which indicated more toluene was adsorbed on the catalysts. However, the absence of ozone would lead to a high temperature sintering after the reaction, which resulted in a decrease in the specific surface area of the catalysts and less adsorbed toluene. The carboxylate vibrational peak could be observed at 1678 cm^−1^^52^, which suggested that toluene might be oxidized into benzaldehyde. The vibrational peaks of carboxylates could be observed at 1593 cm^−1^^53^, and the C–O stretching vibration of alcohol and phenolic at 1269 cm^−1^. This suggested that toluene might be oxidized to C–O– containing alcohols and phenols, and COO– containing intermediates. The intensity of these two characteristic peaks after the ozone-catalyzed oxidation tended to be greater than those without ozone, indicating that the addition of ozone improved the oxidation of toluene. The peak at 2360 cm^−1^ was thought to be CO_2_, which also suggested that ozone-catalyzed oxidation leaded to a higher conversion rate of toluene.

The FTIR spectra of Mn_2_Co_3_/Cord catalyst was shown in Fig. 4b including the catalytic oxidation of toluene with and without ozone. The benzene ring skeleton of toluene was observed at 1455 cm^−1^, the carboxylate vibrational peak at 1678 cm^−1^, the vibrational peaks of carboxylates at 1593 cm^−1^ and the C–O stretching vibration of alcohol and phenolic at 1260 cm^−1^. O–H telescopic absorption peak was observed as well at 3438 cm^−1^ with the presence of ozone. In addition, Mn_3_Co_2_/Cord catalyst showed a bending vibration of the C–H bond on the benzene ring at 1175 cm^−1^ with ozone but not without ozone. It might be similar to other characteristic peaks and could not be separated. The peak of CO_2_ was not detected under ozone conditions due to the high catalytic activity of the Mn_2_Co_3_/Cord catalyst, and much more toluene and ozone occupied the surface of the catalyst so that CO_2_ was not adsorbed on the surface. Similar with Fig. 4a and b, the infrared spectra of Cu_3_Co_2_/Cord catalyst in Fig. 4c showed that the benzene ring skeleton of toluene was observed at 1455 cm^−1^, the carboxylate vibrational peak at 1677 cm^−1^, the vibrational peaks of carboxylates at 1590 cm^−1^ and the C–O stretching vibration of alcohol and phenolic at 1269 cm^−1^ under both conditions with and without ozone. In the presence of ozone, the catalyst showed an extra peak at 3445 cm^−1^, which was thought to be O–H telescopic absorption peak^54^.

After analyzing the infrared spectra of these three catalysts, the reaction process of ozone co-catalyzed oxidation of toluene could presumed. First, O· was produced by the catalytic decomposition of ozone, then the toluene was oxidized into benzyl alcohol. The O–H on the methyl group of benzyl alcohol was broken down to form ·OH, which together with O· oxidized benzyl alcohol into benzaldehyde. After the following deep oxidation, they were transformed into alcohols, aldehydes and acids with low carbon chains, and finally oxidized completely into CO_2_ and H_2_O.

### Experiments of toluene degradation by ozone and catalysts

#### Effects of active components ratio on toluene degradation

**Figure 5:**
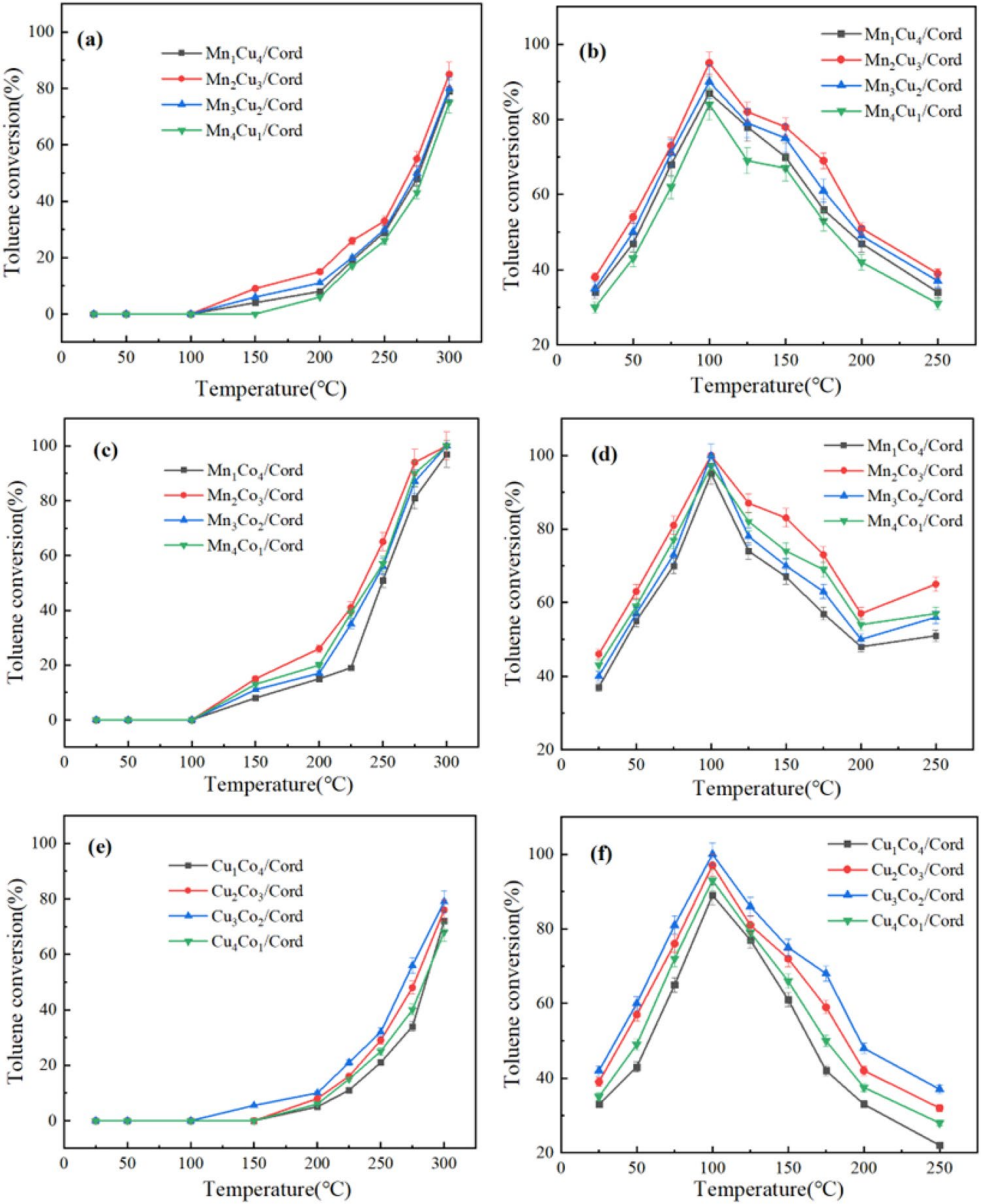
Degradation rate of toluene under different bimetal ratios (**a**) Mn_x_Cu_5−x_/Cord without ozone; (**b**) Mn_x_Cu_5−x_/Cord with ozone; (**c**) Mn_x_Co_5−x_/Cord without ozone; (**d**) Mn_x_Co_5−x_/Cord with ozone; (**e**) Cu_x_Co_5−x_/Cord without ozone; (**f**) Cu_x_Co_5−x_/Cord with ozone.

The bimetal ratios of active components on toluene degradation were studied under the conditions that the initial toluene concentration was 775.6 mg/m^3^, ozone concentration was set to be 3.0 g/m^3^ if ozone presented, and the space velocity was controlled at 12,000 h^−1^. Mn_x_Cu_5−x_/Cord, Mn_x_Co_5−x_/Cord and Cu_x_Co_5−x_/Cord monolithic catalysts were investigated. Both presence and absence of ozone were covered, and the results were shown in Fig. 5.

The degradation rate of toluene by bimetal monolithic catalysts without ozone could be seen in Fig. 5a, c and e. The degradation rate of toluene for all the three kinds of bimetal monolithic catalysts in four ratios all increased with the rising reaction temperature. The degradation started at around 100 °C, and most of the catalysts could completely degrade all the toluene at 300 °C. The catalytic activity of Mn_2_Cu_3_/Cord in Fig. 4a, Mn_2_Co_3_/Cord in Fig. 4b and Cu_3_Co_2_/Cord in Fig. 4c were the best, which was consistent with the previous XRD results in Fig. 2. Figure 5b, d and f showed the degradation rate of toluene with ozone, and the results indicated that the addition of ozone did not change the activity order of the four catalysts. When the reaction temperature ranged from 25 to 100 °C, the degradation rate of toluene under the four catalysts increased with the rising reaction temperature. When the reaction temperature exceeded 100 °C, the degradation rate of toluene decreased with the rising temperature. Since the chemical stability of ozone was weak under high temperature, the decrease in toluene degradation rate was mainly due to the decreasing concentration of ozone with rising temperature. Therefore, the performance of all the Mn_x_Cu_5−x_/Cord, Mn_x_Co_5−x_/Cord and Cu_x_Co_5−x_/Cord monolithic catalysts on toluene degradation reached the highest at 100 °C. The toluene degradation rates from Mn_1_Co_4_/Cord, Mn_2_Co_3_/Cord, Mn_3_Co_2_/Cord and Mn_4_Co_1_/Cord were 95, 100, 99 and 97%, respectively.

#### Effects of ozone concentrations on toluene degradation

**Figure 6:**
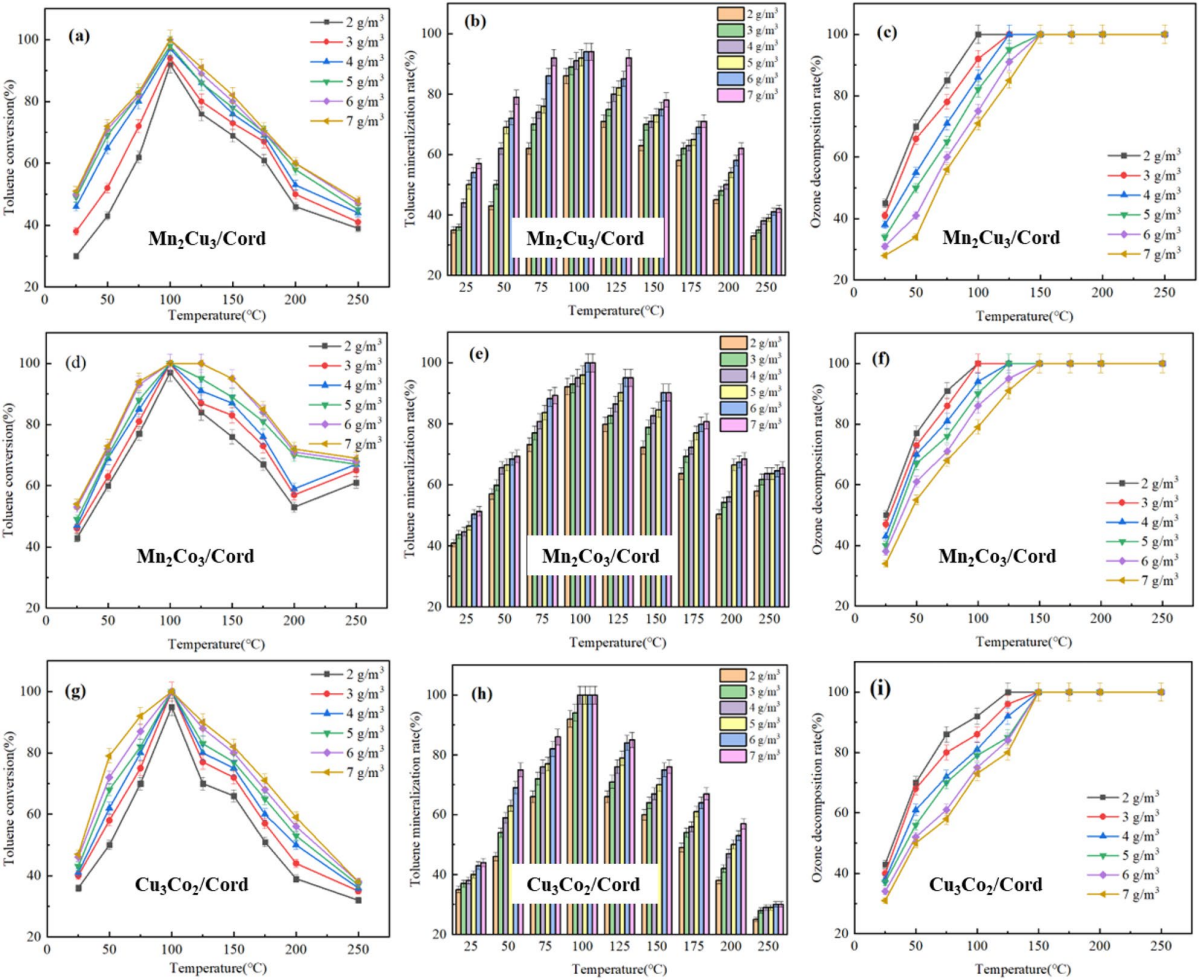
Effect of different ozone concentrations on toluene degradation through bimetal/Cord.

To find out the effects of ozone concentrations on toluene degradation by bimetal/Cord monolithic catalysts, the experimental conditions was set under the ozone concentration gradient of 2.0–7.0 g/m^3^. Mn_2_Cu_3_/Cord, Mn_2_Co_3_/Cord and Cu_3_Co_2_/Cord were selected as the catalysts, the initial concentration of toluene was 775.6 mg/m^3^ and space velocity was set 12,000 h^−1^. Results of the experiment were shown in Fig. 6.

The toluene conversion rates of Mn_2_Cu_3_/Cord, Mn_2_Co_3_/Cord and Cu_3_Co_2_/Cord catalysts under different ozone concentrations were shown in Fig. 6a, d and g, respectively. The conversion rates of toluene increased with the rising ozone concentration, and toluene could be completely degraded at 100 °C. However, when the ozone concentration was greater than 6.0 g/m^3^, the toluene degradation rate increased very little with the rising ozone concentration. This phenomenon might be due to the catalytic ability of bimetal/Cord catalysts reaching their limits when the ozone concentration rose up to 6.0 g/m^3^. Meanwhile, the active oxygen species produced by the catalyzed ozone reached the maximum value. Therefore, when the ozone concentration continued to rise up, the degradation rate of toluene did not increase any more.

The toluene mineralization rate of the Mn_2_Cu_3_/Cord, Mn_2_Co_3_/Cord and Cu_3_Co_2_/Cord catalysts under different ozone concentrations were shown in Fig. 6b, e and h, respectively. The CO_2_ production rate increased with the rising ozone concentration, and toluene mineralization rate could reach more than 90% when the temperature was 100 °C. It could be seen that when the ozone concentration increased, the degradation rates of toluene also increased, which might produce more CO_2_. In addition, when the ozone concentration increased, a small amount of intermediate products generated by the degradation of toluene would react with excess ozone to generate free radicals like ·OH. Meanwhile, O^**·**^ and ·OH radicals could oxidize intermediate products such as benzoic acid into products with multiple carbon-based bonds and chain olefins, and finally oxidized into H_2_O and CO_2_, thus increasing the mineralization rate.

Figure 6c, f and i showed the decomposition rate of ozone under different ozone concentrations with Mn_2_Cu_3_/Cord, Mn_2_Co_3_/Cord and Cu_3_Co_2_/Cord catalysts, respectively. The ozone decomposition rate decreased with the rising ozone concentration. The ozone decomposition rate reached 100% at 125 °C, when the ozone concentration ranged from 2.0 to 5.0 g/m^3^. It reached 100% at 150 °C when the ozone concentration was 6.0 and 7.0 g/m^3^. This was mainly because the ability of the catalyst to catalyze ozone was limited, and the increase of ozone concentration would reduce its degradation rate at the same temperature. When the ozone concentration rose up to 6.0 g/m^3^, the maximum amount of ozone that the catalyst could catalyze had exceeded. Therefore, the degradation rate could not reach 100% at 125 °C. When the reaction temperature continued to rise up, the catalytic ability of the catalyst increased, and the decomposition of ozone itself accelerated with the rising reaction temperature. Therefore, the ozone decomposition rate could reach 100% at 150 °C.

#### Effects of space velocity on toluene degradation

**Figure 7:**
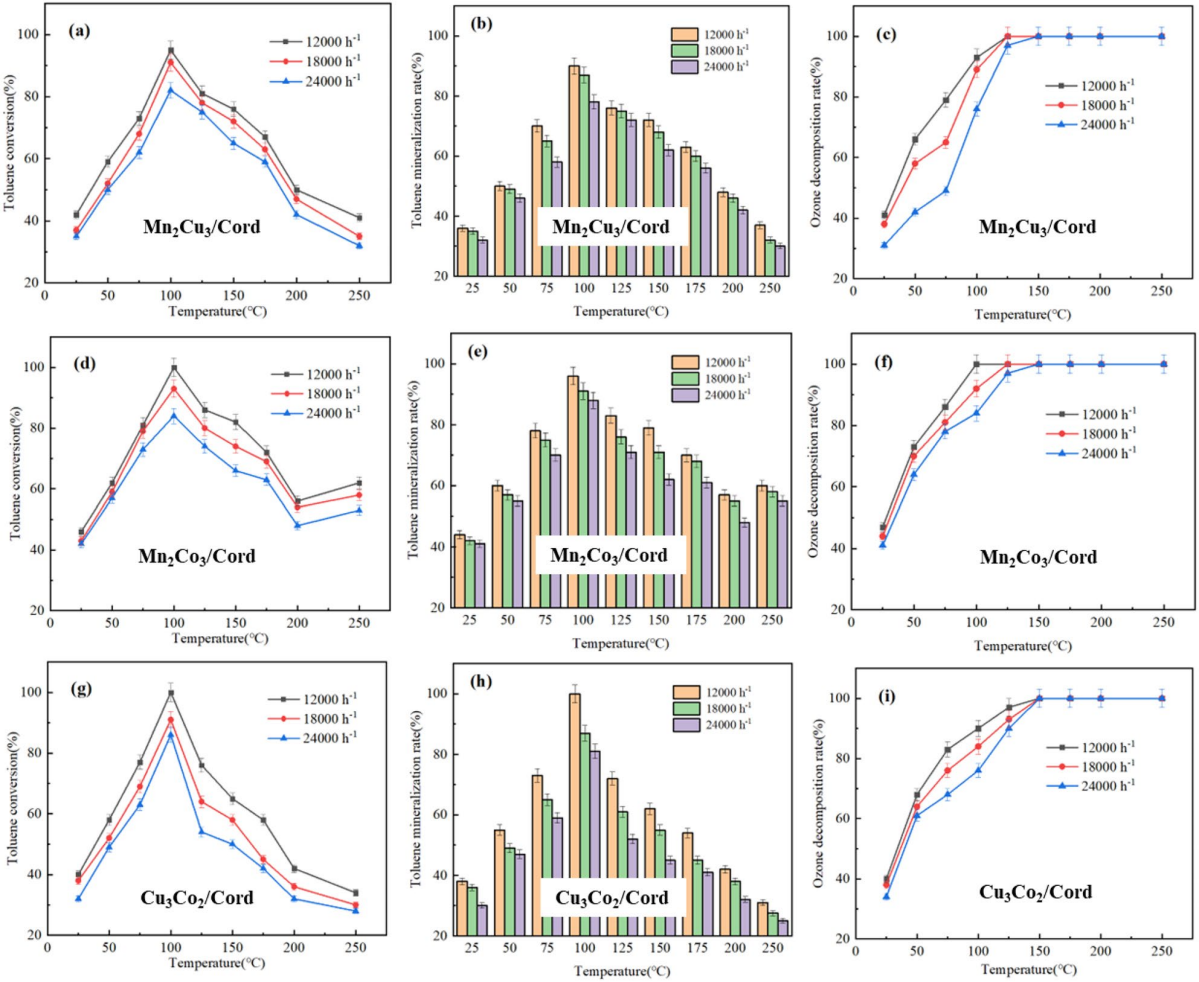
Effect of space velocities on toluene degradation through bimetal/Cord.

Space velocity was another important factor that directly affect the degradation rate of toluene. The gradient of space velocity was set to be 12,000, 18,000 and 24,000 h^−1^. Mn_2_Cu_3_/Cord, Mn_2_Co_3_/Cord and Cu_3_Co_2_/Cord were selected as the catalysts, ozone concentration was 3.0 g/m^3^ and the initial concentration of toluene was 775.6 mg/m^3^. Results of the experiments were shown in Fig. 7.

The conversion rates of toluene at different space velocities could be seen in Fig. 7a, d and g. It decreased with the rising space velocity, because the number of toluene molecules passing through the Mn_2_Cu_3_/Cord, Mn_2_Co_3_/Cord and Cu_3_Co_2_/Cord catalyst increased per hour, while the active sites on the surface of these catalysts remained the same. Since the amount of toluene oxidized by active oxide per hour was settled, the degradation rate of toluene would decrease with the increase of space velocity under the same experimental conditions. Figure 7b, e and h showed the change of toluene mineralization rate at different space velocities. The mineralization rate decreased with the increase of space velocity, because the mineralization ability of the catalyst was related to its own redox ability. When the amount of oxidized toluene decreased, the mineralization rate decreased accordingly. Figure 7c, f and i showed the decomposition rate of ozone at different space velocities. The ozone decomposition rate also decreased with the rising space velocity. When the space velocity increased, the number of ozone molecules passing through the catalyst per hour increased. Since the number of active sites on the surface of the catalyst remained unchanged, the number of active oxygen atoms generated by catalytic ozone per hour was also constant, thereby reducing the decomposition rate of ozone. In addition, when the space velocity increased, the residence time of ozone in the reactor decreased, and the effect of the reaction temperature would be also reduced.

Mn_2_Co_3_/Cord and Cu_3_Co_2_/Cord in this study showed great advantages under low temperature when compared with some representative studies on toluene conversion as shown in Table 2. This mainly because O_3_ addition Effectively increased the content of oxidative free radicals, which helped the staged process of toluene oxidation.

**Table 2:** Representative studies on toluene conversion with catalysts.

No	Catalysts	Toluene conversion rate (%)	Temperature	References
1	Mn_2_Cu_3_/Cord + O_3_	95	100 °C	This study
2	Mn_2_Co_3_/Cord + O_3_	100	100 °C	This study
3	Cu_3_Co_2_/Cord + O_3_	100	100 °C	This study
4	Pd-catalytic Electro-Fenton	71	Ambient temperature	Liao et al.^55^
5	Ag/MnO_2_-cordierite	90	275 °C	Zhu et al.^56^
6	Pt–Pd/MnO_2_	100	175 °C	He et al.^5^
7	Pd-Fe/modified red mud	100	200 °C	Fang et al.^42^
8	Co_1_Cu_1_O_x_-MOF	90	208 °C	Lei et al.^46^
9	Pt-La_1−x_Sr_x_MnO_3_	90	166 °C	Li et al.^48^
10	α-MnO_2_	90	226 °C	Min et al.^43^
11	Cu/SmMn_2_O_5_	90	206 °C	Shen et al.^16^
12	Ag/TiO_2_ + UV	88	180 °C	Dursun et al. ^32^

### Kinetics and mechanism of toluene oxidation by O_3_ with catalysts

#### Kinetics of toluene oxidation

**Figure 8:**
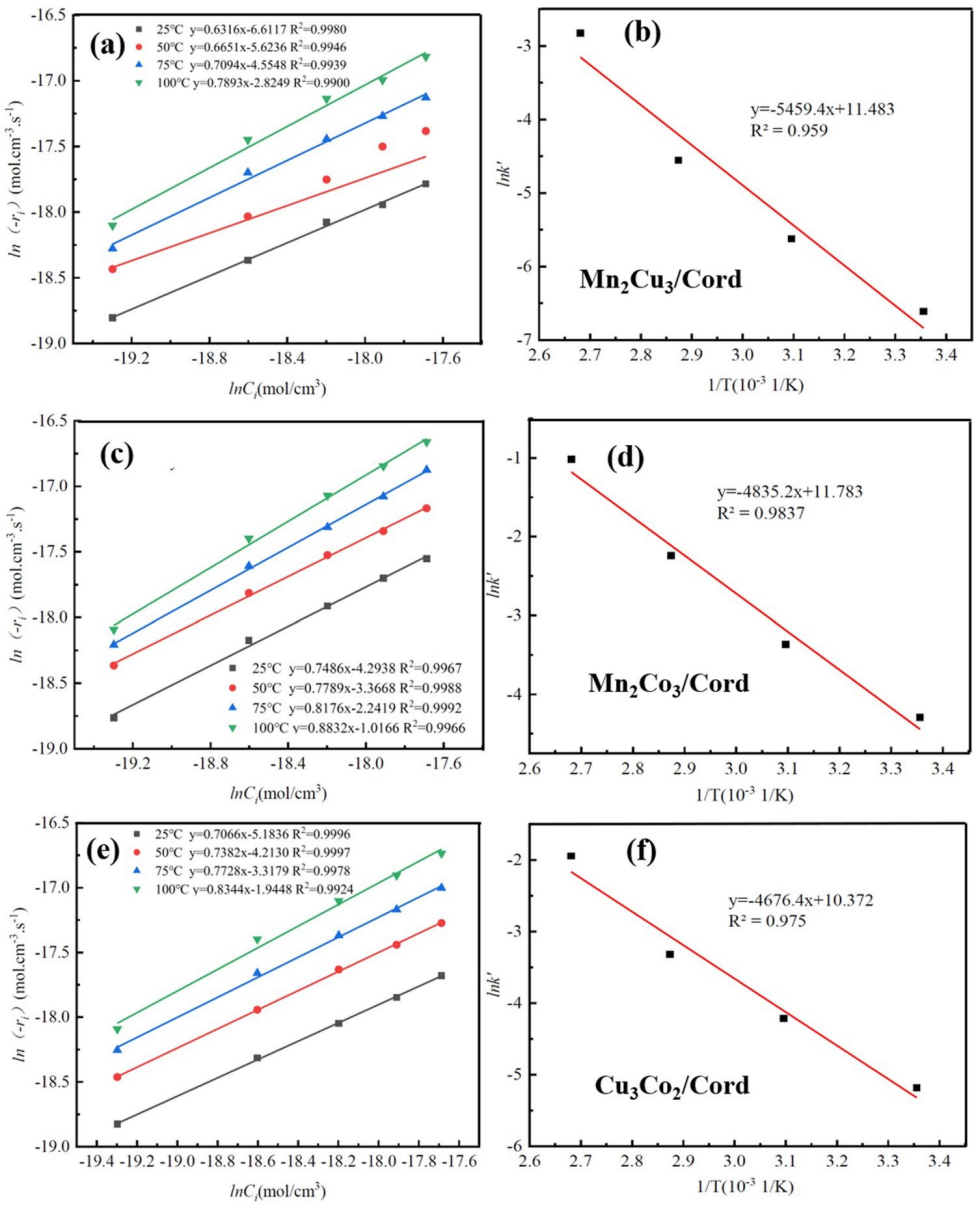
Kinetics of toluene oxidation by O_3_ with catalysts.

In the presence of ozone, toluene degradation rate firstly increased and then decreased with the rising temperature. Kinetics of toluene oxidation by O_3_ with Mn_2_Cu_3_/Cord, Mn_2_Co_3_/Cord and Cu_3_Co_2_/Cord catalysts were discussed in this section. The experimental conditions were set as follows: ozone concentration of 3.0 g/m^3^, space velocity of 12,000 h^−1^. Initial toluene concentrations were selected as 383.3, 766.5, 1149.8, 1533.0, and 1916.3 mg/m^3^, and the reaction temperatures were selected as 25, 50, 75, and 100 °C. The toluene degradation rates at different reaction temperatures were shown in Tables s3–5. The reaction rate r_A_ could be calculated when taking toluene degradation rate into Eq. (3), and the results were shown in Tables s6–8. A linear fit was performed by taking *l*(*r*_*i*_) as the vertical coordinate and *lnC*_*i*_ as the horizontal coordinate. The results of the simulation are shown in Fig. 8a, c and e.

3 $$r_{A} = \frac{{F_{A0} }}{{V_{R} }}\frac{{C^{\prime} - C_{0} }}{{C_{0} }}$$

where r_A_ was the reaction rate of component A, mol/(cm^3^·s); F_A0_ was the moles of component A flowing into the reactor per unit time, mol/s; V_R_ was the volume of reactor, cm^3^; C′ was the concentration of component A at the outlet, mol/cm^3^; C_0_ was the concentration of component A at the inlet, mol/cm^3^.

Figure 8a, c and e show that the R^2^ of all four regression lines were above 0.99, indicating a good fitness. Then the values of reaction orders n and observed rate constants *k*′ were derived according to Eq. (4), and the results were shown in Tables s9–11. Finally, a linear fit of *lnk* and T^−1^ was performed according to the Arrhenius equation as shown in Eqs. (5) and (6), and the results were shown in Fig. 8b, d and f. The slope of the obtained straight line could be calculated as the reaction activation energy E_a_, and the intercept could be calculated as the pre-exponential factor A. As seen in the figures, R^2^ > 0.9 suggested that this kinetic model could be used to investigate the kinetic behavior of the ozone-catalyzed oxidation of toluene. After calculation, the activation energy of Mn_2_Cu_3_/Cord E_a1_ = 45.39 kJ/mol, Mn_2_Co_3_/Cord E_a2_ = 40.20 kJ/mol, Cu_3_Co_2_/Cord E_a3_ = 3.8.88 kJ/mol. Cu_3_Co_2_/Cord catalyst was the easiest for ozone-catalyzed oxidation reactions among these three.

4 $$r_{VOCs} = \frac{{dC_{VOCs} }}{dt} = kC_{VOCs}^{n} C_{{O_{3} }}^{m} = k^{\prime}C_{VOCs}^{n}$$

where r_VOCs_ was the reaction rate of VOCs, mol/(cm^3^ s); C_VOCs_ was the concentration of VOCs, mol/cm^3^; C_O3_ was the concentration of O_3_, mol/cm^3^; m and n were the reaction orders of O_3_ and VOCs; k was the reaction rate constants; k′ was the observed rate constants, k′ = k $C_{{O_{3} }}^{m}$.

5 $$k = Ae^{{ - \frac{{E_{a} }}{RT}}}$$

6 $$\ln k = - \frac{{E_{a} }}{RT} + \ln A$$

where A was the pre-exponential factor, s^−1^; E_a_ was the activation energy, kJ/mol; R was the ideal gas constant, 8.31 × 10^–3^ kJ/(mol·K); T was the reaction temperature, K.

#### Toluene oxidation mechanism by O_3_ with catalysts

**Figure 9:**
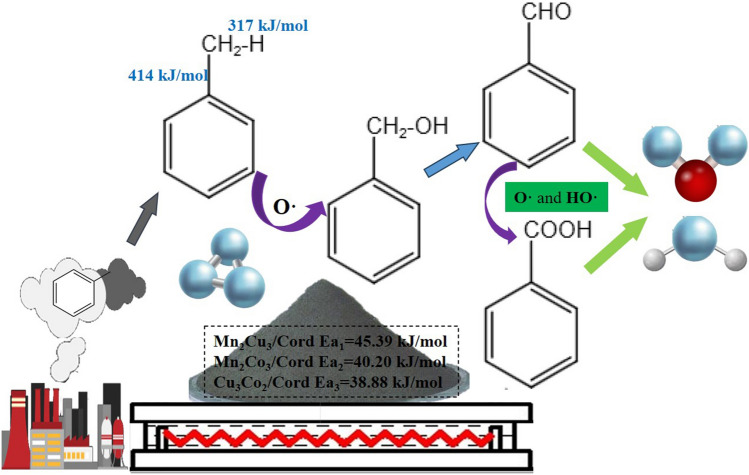
Schematic illustration of toluene oxidation mechanism by O_3_ with catalysts.

Ozone was first decomposed into O**·** in the presence of catalysts, which broke down the C–H bond of the methyl group on toluene through oxidation, and then benzyl alcohol was produced. The -OH of benzyl alcohol was broken down to form HO·, along with O· generated by ozone decomposition, these two oxidative free radicals helped oxidize benzyl alcohol into benzaldehyde. Part of the benzaldehyde was further oxidized into benzoic acid, and the remaining benzaldehyde together with the produced benzoic acid was deeply oxidized into low-carbon chain alcohols, aldehydes and acids, and they were finally completely mineralized in to CO_2_ and H_2_O. Schematic illustration of toluene oxidation mechanism was shown in Fig. 9.

## Conclusions

Experimental study on the synergistic oxidation of toluene by ozone and bimetal/Cord monolithic catalysts has been carried out in this research. Effects of active component ratio, ozone addition and space velocity on toluene degradation were investigated, and characteristics on Mn_x_Cu_5−x_/Cord, Mn_x_Co_5−x_/Cord and Cu_x_Co_5−x_/Cord (x = 1, 2, 3, 4) were conducted. Conclusions could be drawn as follows:

1. The characterizations of Mn_x_Co_1−x_/Cordierite catalysts results showed that the addition of ozone could effectively avoid the high temperature sintering of the catalyst, and the specific surface area and crystal structure of the catalyst had little change before and after the reaction. The spinel diffraction peak intensity of Mn_2_Co_3_/Cord catalyst was the highest, which suggested its best catalytic activity. Mn_2_Co_3_/Cord could completely degrade toluene at 300 °C without ozone, while toluene degradation rate reached 100% only at 100 °C after adding ozone.
2. The degradation rate of toluene increased with rising ozone concentration, because intermediate products generated by toluene degradation could react with excess ozone to generate free radicals like ·OH, which would improve the toluene mineralization rate of the Mn_2_Co_3_/Cord catalyst. Toluene degradation rate decreased with the rising space velocity due to the increasing amount of toluene molecules passing through the Mn_2_Co_3_/Cord catalyst under the limited active sites on the surface of the catalyst.
3. Bimetal/cord monolithic catalysts in this study showed great advantages under low temperature when compared with some representative studies. Combined with economic benefit and environmental protection concept, the reaction temperature for the synergistic oxidation of toluene by bimetal/Cord monolithic catalysts and ozone could be selected as 100 °C, space velocity set to be 12,000 h^−1^ and ozone concentration was 3.0 g/m^3^. Under these optimal conditions, the degradation rate of 766.5 mg/m^3^ toluene could reach 100%, and the toluene mineralization rate and ozone decomposition rate were 96 and 100%, respectively. The findings in this study would provide theoretical basis and technical reference for the toluene treatment in industrial applications.

### Supplementary Information

Supplementary Information.
