# Supplementary material for: Synergistic oxidation of toluene through bimetal/cordierite monolithic catalysts with ozone
Source: Sci Rep. 2024 Mar 26;14:7203. doi: 10.1038/s41598-024-58026-6 (PMC10965967; doi:10.1038/s41598-024-58026-6)
Supplement: Supplementary file 1 — Supplementary Information. [file 41598_2024_58026_MOESM1_ESM.docx]

Synergistic Oxidation of Toluene through Bimetal/Cordierite Monolithic Catalysts with Ozone

Xiaojian Wang ^a, b^, Xiaomin Peng ^a, c^, Quanzhong Zhao ^d^, Jinxing Mi ^e^, Huating Jiang ^a^, Shengli Li ^a, c^, Hui Hu ^a, c^, Hao Huang ^a, c *^

^a^ *School of Environmental Science and Engineering, Huazhong University of Science and Technology, Wuhan 430074, P.R. China*

^b^ *Shanghai Tobacco Group Co. LTD, Shanghai 200082, P.R. China*

^c^ *Hubei Key Laboratory of Multi-media Pollution Cooperative Control in Yangtze Basin, School of Environmental Science and Engineering, Huazhong University of Science and Technology, Wuhan 430074, P.R. China*

^d^ *Inner Mongolia Power Research Institute Branch, Inner Mongolia Power (Group) Co.,Ltd., Hohhot 010020, P.R. China*

^e^ *State Key Joint Laboratory of Environment Simulation and Pollution Control, School of Environment, Tsinghua University, Beijing 100084, P. R. China*

Corresponding author: Hao Huang (huanghao@hust.edu.cn).

# 1. Textural properties of the Mn_x_Cu_5-x_/Cord and Cu_x_Co_5-x_/Cord

Textural properties of Mn_x_Cu_5-x_/Cord and Cu_x_Co_5-x_/Cord were listed in Table s1 and s2, including before and after catalytic oxidation reactions as well as with and without ozone.

**Table s1 Textural properties of Mn_x_Cu_5-x_/Cord**

| Samples | a: Before reaction.  b: After reaction without ozone.  c: After reaction with ozone. | BET surface area  （m^2^/g） | Pore volume  （cm^3^/g） | Average pore diameter  （nm） |
| --- | --- | --- | --- | --- |
| Cord | a | 1.0686 | 0.0032 | 11.9981 |
| Mn_1_Cu_4_/Cord | a | 2.7305 | 0.0086 | 22.2189 |
|  | b | 2.2485 | 0.0067 | 12.5687 |
|  | c | 3.167 | 0.0211 | 26.7726 |
| Mn_2_Cu_3_/Cord | a | 3.2202 | 0.0147 | 18.2654 |
|  | b | 2.4712 | 0.0105 | 17.1234 |
|  | c | 3.167 | 0.0211 | 26.7726 |
| Mn_3_Cu_2_/Cord | a | 2.9761 | 0.0169 | 22.7587 |
|  | b | 2.6752 | 0.0107 | 16.0096 |
|  | c | 3.1528 | 0.0174 | 17.599 |
| Mn_4_Cu_1_/Cord | a | 2.6031 | 0.0136 | 13.218 |
|  | b | 2.1449 | 0.0052 | 10.4662 |
|  | c | 2.6952 | 0.0097 | 16.4996 |

The top BET surface area of Mn_x_Cu_5-x_/Cord reached 3.2202 m^2^/g at the ratio of Mn:Cu=2:3.

**Table s2 Textural properties of Cu_x_Co_5-x_/Cord**

| Samples | a: Before reaction.  b: After reaction without ozone.  c: After reaction with ozone. | BET surface area  （m^2^/g） | Pore volume  （cm^3^/g） | Average pore diameter  （nm） |
| --- | --- | --- | --- | --- |
| Cord | a | 1.0732 | 0.00302 | 11.8963 |
| Cu_1_Co_4_/Cord | a | 1.735 | 0.0081 | 27.4167 |
|  | b | 1.445 | 0.0053 | 37.0993 |
|  | c | 1.8234 | 0.009 | 28.2578 |
| Cu_2_Co_3_/Cord | a | 3.7366 | 0.0144 | 14.7343 |
|  | b | 3.2952 | 0.0121 | 17.2697 |
|  | c | 3.7105 | 0.0137 | 14.8054 |
| Cu_3_Co_2_/Cord | a | 3.8408 | 0.0131 | 13.7265 |
|  | b | 3.4586 | 0.0127 | 16.1388 |
|  | c | 3.7942 | 0.0148 | 13.9482 |
| Cu_4_Co_1_/Cord | a | 2.6345 | 0.0107 | 11.7688 |
|  | b | 1.5232 | 0.0078 | 20.5883 |
|  | c | 2.7032 | 0.0106 | 11.6542 |

The top BET surface area of Cu_x_Co_5-x_/Cord reached 3.8408 m^2^/g at the ratio of Co:Cu=2:3.

# 2. SEM images of original cordierite

The surface of the blank cordierite was smooth and flat as shown in Fig. 1s, and some small particles might be caused by inadequate grinding carriers.


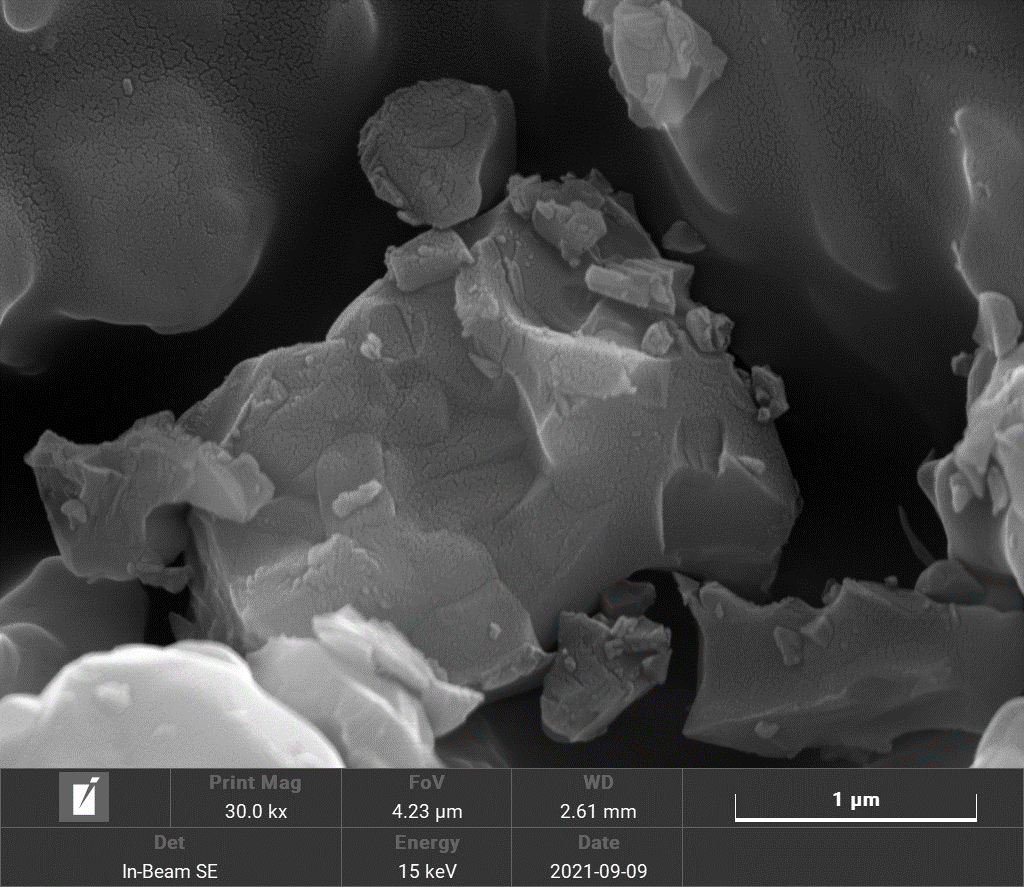


Fig. 1s SEM images of blank cordierite before reactions

# 3. Data of the reaction kinetics

Toluene degradation by Mn_2_Cu_3_/Cord, Mn_2_Co_3_/Cord and Cu_3_Co_2_/Cord catalysts with ozone under different temperatures were shown in Table s3-5, respectively. It could be seen that toluene degradation rate increased with the rising temperature at the same toluene concentration. Toluene degradation rate decreased with the rising initial toluene concentration at the same temperature, because the number of toluene molecules increased with increasing toluene concentration, which made the toluene loaded per unit of catalyst bed increased. Since the transforming capacity of monolithic catalysts stayed the same, the degradation rate decreased with the rising toluene concentration.

Table s3 degradation rate of toluene by Mn_2_Cu_3_/Cord under different temperatures

| Initial concentration of toluene (10^-9^ mol/cm^3^) | Toluene degradation rate | | | |
| --- | --- | --- | --- | --- |
|  | 25 °C | 50 °C | 75 °C | 100 °C |
| 4.16 | 49% | 71% | 83% | 99% |
| 8.34 | 38% | 53% | 74% | 95% |
| 12.50 | 34% | 47% | 64% | 87% |
| 16.66 | 29% | 45% | 57% | 75% |
| 20.82 | 27% | 40% | 52% | 71% |

Table s4 degradation rate of toluene by Mn_2_Co_3_/Cord under different temperatures

| Initial concentration of toluene (10^-9^ mol/cm^3^) | Toluene degradation rate | | | |
| --- | --- | --- | --- | --- |
|  | 25 °C | 50 °C | 25 °C | 100 °C |
| 4.16 | 51% | 73% | 89% | 100% |
| 8.34 | 46% | 63% | 81% | 100% |
| 12.50 | 40% | 59% | 73% | 93% |
| 16.66 | 37% | 55% | 69% | 87% |
| 20.82 | 34% | 50% | 67% | 83% |

Table s5 degradation rate of toluene by Cu_3_Co_2_/Cord under different temperatures

| Initial concentration of toluene (10^-9^ mol/cm^3^) | Toluene degradation rate | | | |
| --- | --- | --- | --- | --- |
|  | 25 °C | 50 °C | 25 °C | 100 °C |
| 4.16 | 48% | 69% | 85% | 100% |
| 8.34 | 40% | 58% | 77% | 100% |
| 12.50 | 35% | 53% | 69% | 90% |
| 16.66 | 31% | 48% | 63% | 82% |
| 20.82 | 30% | 45% | 59% | 77% |

Based on the toluene degradation rates in Table s3-5, the reaction rates could be calculated by putting them into Eq. 3, and the results were shown in Table s6-8. It could be seen that the reaction rates of ozone-catalyzed oxidation increased both with the initial concentration of toluene and reaction temperature.

Table s6 reaction rate of toluene degradation by Mn_2_Cu_3_/Cord under different temperatures

| Initial concentration of toluene (10^-9^ mol/cm^3^) | Reaction rate r_i_ (10^-9^ mol/cm^3^·s) | | | |
| --- | --- | --- | --- | --- |
|  | 25 °C | 50 °C | 25 °C | 100 °C |
| 4.16 | 6.81 | 9.86 | 11.53 | 13.76 |
| 8.34 | 10.56 | 14.73 | 20.57 | 26.41 |
| 12.50 | 14.11 | 19.5 | 26.56 | 36.1 |
| 16.66 | 16.09 | 24.97 | 31.63 | 41.62 |
| 20.82 | 18.9 | 28 | 36.4 | 49.7 |

Table s7 reaction rate of toluene degradation by Mn_2_Co_3_/Cord under different temperatures

| Initial concentration of toluene (10^-9^ mol/cm^3^) | Reaction rate r_i_ (10^-9^ mol/cm^3^·s) | | | |
| --- | --- | --- | --- | --- |
|  | 25 °C | 50 °C | 25 °C | 100 °C |
| 4.16 | 7.08 | 10.11 | 12.37 | 13.9 |
| 8.34 | 12.78 | 17.51 | 22.51 | 27.8 |
| 12.5 | 16.6 | 24.48 | 30.29 | 38.59 |
| 16.66 | 20.53 | 30.52 | 38.29 | 48.28 |
| 20.82 | 23.8 | 35 | 46.9 | 58.1 |

Table s8 reaction rate of toluene degradation by Cu_3_Co_2_/Cord under different temperatures

| Initial concentration of toluene (10^-9^ mol/cm^3^) | Reaction rate r_i_ (10^-9^ mol/cm^3^·s) | | | |
| --- | --- | --- | --- | --- |
|  | 25 °C | 50 °C | 25 °C | 100 °C |
| 4.16 | 6.67 | 9.59 | 11.81 | 13.9 |
| 8.34 | 11.12 | 16.12 | 21.4 | 27.8 |
| 12.5 | 14.52 | 21.99 | 28.63 | 37.35 |
| 16.66 | 17.76 | 26.64 | 34.96 | 45.51 |
| 20.82 | 21 | 31.5 | 41.3 | 53.9 |

A linear fit was performed by using 𝑙𝑛 𝑟_𝑖_ as the vertical coordinate and 𝑙𝑛 𝐶𝑖 as the horizontal coordinate. The results of the simulation were shown in Fig. 8. It showed that the R^2^ of all four regression lines were above 0.99, indicating a good fit. Then the values of reaction orders n and the reaction rate constant 𝑘′ were derived according to Eq. 5. The results were shown in Table s9-11.

Table s9 reaction order and reaction rate constant of toluene degradation by Mn_2_Cu_3_/Cord

| Temperature (°C) | Reaction order n | Reaction rate constant k' (10^-3^ s-^1^) |
| --- | --- | --- |
| 25 | 0.6316 | 1.345 |
| 50 | 0.6651 | 3.612 |
| 75 | 0.7094 | 10.517 |
| 100 | 0.7893 | 59.314 |

Table s10 reaction order and reaction rate constant of toluene degradation by Mn_2_Co_3_/Cord

| Temperature (°C) | Reaction order n | Reaction rate constant k' (10^-3^ s-^1^) |
| --- | --- | --- |
| 25 | 0.7486 | 1.365 |
| 50 | 0.7789 | 3.45 |
| 75 | 0.8176 | 10.626 |
| 100 | 0.8832 | 33.068 |

Table s11 reaction order and reaction rate constant of toluene degradation by Cu_3_Co_2_/Cord

| Temperature (°C) | Reaction order n | Reaction rate constant k' (10^-3^ s-^1^) |
| --- | --- | --- |
| 25 | 0.7066 | 0.561 |
| 50 | 0.7382 | 1.48 |
| 75 | 0.7728 | 3.623 |
| 100 | 0.8344 | 14.302 |
